# The Effects of Energy Drink Consumption on Cognitive and Physical Performance in Elite *League of Legends* Players

**DOI:** 10.3390/sports7090196

**Published:** 2019-08-22

**Authors:** Casey J. Thomas, Jeffrey Rothschild, Conrad P. Earnest, Aaron Blaisdell

**Affiliations:** 1 School of Kinesiology and Nutritional Science, California State University Los Angeles, Los Angeles, CA 90032, USA; 2 Athletics Department, University of California, Los Angeles, Los Angeles, CA 90095, USA; 3 TriFit Performance Center, Santa Monica, CA 90404, USA; 4 Department of Health & Kinesiology, Texas A&M University, College Station, TX 77843, USA; 5 Department of Psychology & Brain Research Institute, University of California, Los Angeles, Los Angeles, CA 90095, USA

**Keywords:** eSports, caffeine, cognition, energy drink, gamer, sports nutrition

## Abstract

To examine the cognitive and physical changes associated with consuming an energy drink concurrent to video gaming, we examined a convenience sample of nine elite League of Legends (LoL) e-sport players (21 ± 2 y, BMI 25.6 ± 3.4 kg/m^2^) consuming an energy drink (Reload^TM^) or placebo (Placebo) in a randomized, double-blind, placebo-controlled cross-over trial. Participants completed the same test battery prior to treatment consumption and after playing each of three competitive LoL games. Primary outcomes included measures of attention (Erikson Flanker Test), reaction time (Go/No-Go test) and working memory (n-back test). Secondary outcomes examined fatigue (hand grip strength and finger tap speed). Statistical analysis was performed by repeated-measures analysis of variance (RM-ANOVA) and reported as the mean (standard deviation [SD]) or mean change (95% confidence interval [CI]). Participants reported sleeping 8.1 (1.2) h/night, playing LoL 10.3 (2.1) h/d, playing other video games 1.8 (2.8) h/d, and exercising 4.2 (1.7) times per week. Overall, we observed no significant time, group, or group-by-time interactions for any measured performance index with the exception of a significant improvement for the n-back test, where the Reload group demonstrated a significant within-group improvement: Reload [−171 ms (95% CI, −327.91, −14.09), *p* < 0.004], Placebo [−92 ms (95% CI, −213.63, 29.63)]. However, no between-group differences were noted (38.50 ms, 95% CI, −141.89, 64.89, *p* = 0.803). Our findings suggest that elite eSport athletes do not demonstrate a mental or physical improvement in performance relative to the treatment supplement or indices measured in this study.

## 1. Introduction

Electronic sports (video games or eSports) have become increasingly popular throughout the world and have grown into a multi-billion-dollar industry that can act as both entertainment and a form of serious competition. In 2014, more people watched the League of Legends (LoL) World Championship (27 million) than the World Series (23.5 million), NBA Finals (17.9 million), and Stanley Cup (6 million) [1]. Due to the rapid growth and popularity, universities have begun adding eSports to their athletic departments, with several providing scholarships [2]. Currently, LoL is one of the most popular video games, with over 100 million monthly active players [3].

League of Legends is an action strategy game that places two teams of five individuals against one another, with the objective of destroying the opponent’s base. Individual games last less than one hour on average and professional competitions are played in a best-of-three or best-of-five game series. Unlike other sports in which physical traits are key to success, success in eSports is primarily related to mental functioning and ability relative to individual fluid intelligence, coupled with the group’s collective intelligence [4,5]. Mental fatigue has been defined as a change in a person’s psychophysiological state that occurs during or following sustained periods of cognitive activity [6] and is characterized by a decrease in attentional ability [7,8]. This decrease may lead to compromised performance in a variety of cognitive tasks. Therefore, it is reasonable to postulate that mental fatigue is a key component to eSports performance, as players are required to sustain peak cognitive performance, as well as motor behavioral tasks, for several hours of competition. However, no study to date has examined if eSport competitors are affected by cognitive fatigue while gaming.

The competitive nature of eSports has led players to turn to various nootropics, or cognitive enhancing drugs, to gain a performance edge [9]. Several energy drink companies provide sponsorships to eSports teams, and energy drink consumption is pervasive among gamers [2,10]. Energy drinks make claims of attenuating mental fatigue and improving performance by maintaining alertness and wakefulness [11]. One drink that is used during competition and in practice by the members of several professional LoL teams is AI Reload (Reload). Active ingredients in Reload with the potential to affect cognition and/or gaming performance include caffeine and L-theanine (alone and in combination) [12,13,14], phosphatidylserine [15], Nicotinamide Adenine Dinucleotide [in the reduced form as NADH] [16], alpha-glycerylphosphorylcholine (alpha-GPC) [17], and L-Carnitine [18], though studies using this supplement have yet to be performed.

Research into eSports is in its infancy and there is a growing need to investigate the demands on these athletes in order to optimize both player health and competitive performance. While there has been a concerted effort to examine the use of ergogenic aids in other sports, no such studies have been performed in eSports. Several studies have examined the distinguishing traits of elite eSports athletes [19,20,21,22,23]; however, these studies looked at players from games of a different genre and may not directly translate to LoL, which has one of the largest player bases of any current video game [24]. Thus, no evidence-based recommendations can be made for these athletes at present. An additional challenge when researching a game such as LoL is the lack of quantifiable performance metrics. In this study, we examined markers of cognitive function and mental and physiological fatigue in a convenience sample of elite eSport players ingesting an ergogenic supplement or placebo during a simulated competition. We hypothesized that mental fatigue accumulated through three consecutive video games would result in a decline in performance across a battery of cognitive and physical tests, and that the use of an energy drink could reduce any observed decreases.

## 2. Materials and Methods

### 2.1. Study Participants

We are currently unaware of any studies examining eSport performance and were, therefore, unable to perform sample size estimates from prior literature. Accordingly, we recruited and examined a convenience sample of nine male elite LoL players belonging to the same professional team. Therefore, conditions for the LoL component of the study were identical to that of the team’s practice (two teams of five playing in separate rooms against each other, with no one else present, at their team training facility in Beverly Hills, CA, USA). Potential participants were contacted in person, at their training facility, to determine initial interest. Study approval was obtained by California State University, Los Angeles Institutional Review Board (IRB-1116946-1). Prior to study participation, the study investigators reviewed the study with each participant and interested individuals signed a university IRB-approved consent form. The study excluded subjects below 18 years of age and those who have had a history of negative side effects to energy drinks or caffeine.

### 2.2. Study Design

**Figure 1:**
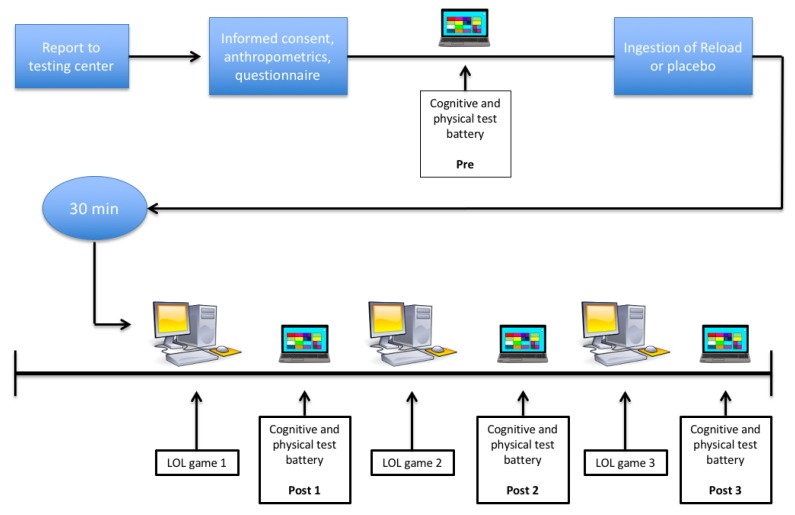
Visual timeline for the study.

A schematic representation of the study procedures is shown in Figure 1. After signing their informed consent, participants were assigned to their respective treatment conditions (described below) in a randomized, double-blind, counter-balanced, cross-over and placebo-controlled manner. Cognitive and physical measures were performed four times per testing day: prior to and after each of three consecutive LoL games. Each game was separated by 15 min for testing procedures and strategic debriefing pertaining to game play. The use of three games was chosen to emulate the best-of-three game format that the players typically compete in. Testing days occurred one week apart, at the same time of day (approximately 13:00) in a well-lit environment. Room temperature was maintained at between 21 and 23 °C. The subjects were instructed to abstain from all caffeine sources on the mornings prior to testing.

### 2.3. Treatment

The treatment condition for this study consisted of an energy drink known as AI Reload (AI Fuels Llc. Santa Monica, CA, USA), which consisted of 12 g Glycerin, 150 mcg Vitamin B12, 70 mg Magnesium, 40 mg Sodium and 1005 mg Proprietary Blend—L-Carnitine, 150 mg Caffeine, L-theanine, Phosphatidylserine, Choline [from alpha-GPC], Nicotinamide Adenine Dinucleotide [reduced form NADH]). The placebo drink beverage was made from Crystal Light™ Pink Lemonade Drink Mix (Crystal Light, Northfield, IL, USA) and consisted of citric acid, potassium citrate, maltodextrin, aspartame, magnesium oxide, and less than 2% of natural flavor, acesulfame potassium, soy lecithin, artificial color, red 40. The beverages were consumed across two visits. To blind participants, placebo and treatment arm beverages were administered in equal volumes. When administering the treatment arm, we mixed in Reload with the Crystal Light™ to mask flavor and color, and participants were unable to correctly guess which drink they had been given. Beverages were consumed after all PRE testing was completed and 30 min prior to the first game of LoL (Figure 1). In order to mimic what is commonly done in practice, the treatment condition consisted of a single serving of AI Reload (118 mL) which included 150 mg caffeine (1.9 ± 0.3 mg per kg body mass).

### 2.4. Cognitive Measures

No standard testing protocols have been established for this population. As such, the assessments conducted were selected because they are frequently used as standard measures in other literature. Subjects completed three cognitive assessments: the Eriksen Flanker Test, the Go/No-go Visual Reaction Time Test, and the Working Memory Test (n-back), using an online source (http://cognitivefun.net/). The Eriksen Flanker Test is used to measure attentional ability, specifically focused attention and the ability to inhibit attention to distractors [25,26,27]. In this test, a target arrow is presented visually to participants. The target arrow is sometimes surrounded by other arrows, facing either congruently or opposite to that of the target arrow, that are to be ignored. All arrows lay horizontally, pointing either left or right. Participants were instructed to press either the right or left arrow key, corresponding to the target arrow direction, as quickly and accurately as possible after presentation. Twenty trials were performed. The following variables were collected and analyzed: congruent mean reaction time (RT, ms), percentage of congruent correct, opposite mean RT (ms), and the percentage of opposite correct. The Go/No-go Visual Reaction Time Test assesses an individual’s ability to inhibit an inappropriate response [28,29]. In this task, two signal cues are presented successively on separate trials. One image corresponds with a ‘go’ stimulus, while the other image corresponds with a ‘no-go’ stimulus. Participants were required to press a key whenever a ‘go’ stimulus was presented and withhold a response (by not pressing the key) whenever a ‘no-go’ stimulus was presented. Participants were instructed to press the key as quickly as possible whenever a ‘go’ stimulus is presented. The percentage of correct responses and mean RT (ms) were collected and analyzed. To assess working memory [30,31], the n-back test presents a random sequence of stimuli to participants. Participants were instructed to click the screen whenever the current stimulus matched the stimulus from two items prior (i.e., a 2-back task). Clicking the screen when the stimulus did not match and failing to click the screen for a matching stimulus were each recorded as an error. The percentage of correct responses and mean RT (ms) were collected and analyzed.

### 2.5. Physical Measures

Subjects completed two physical assessments: a Finger Tap Test and the Handgrip Strength (HGS) Test. The CNS Tap Test phone app (Smudge.IO, Perth, Australia) was used to assess the maximal number of finger taps per 10 s interval using the index finger only, while the other fingers were in contact with the table. Finger tap tests have been used to evaluate co-ordination and hand skill [32]. The best (highest number of finger taps per 10 s interval) of three tests was recorded for each hand during each assessment. Handgrip strength tests have been used to measure fatigue [33]. A handgrip dynamometer (Camry Scale–USA, City of Industry, CA, USA) was used to assess grip strength. The best of three tests was recorded for each hand during each assessment. Body mass was assessed using Seca mBCA 514 (Seca Ltd., Hamburg, Germany).

### 2.6. Questionnaire

In the present study, participants were asked to verbally complete a 9-item questionnaire at the first visit. Three questions pertained to the participants’ demographics: age, sex, and current education level. One question assessed any history of negative side effects to energy drinks or caffeine. The remaining five questions related to participants’ gaming habits (“On average, how much time do you spend playing League of Legends each day?” and “On average, how much time do you spend playing other games each day?”), lifestyle habits (“On average, how many hours of sleep do you get each night?” and “On average, how often do you exercise each week?”), and diet (“Do you feel that you eat a healthy, balanced diet? Why or why not?”).

### 2.7. Statistical Analysis

Primary outcomes were measures of attention (Erikson Flanker Test), reaction time (Go/No-Go test), and working memory (n-back test), while secondary outcomes were measures of fatigue (hand grip strength and finger tap speed) using repeated measures ANOVA and post-hoc testing with Bonferroni adjustments for time, group (i.e., treatment) and group-by-time interactions. Tertiary outcomes were to compare between PRE and POST1 to determine any effects that may have been apparent after one game but not three. Data were analyzed using JASP statistical software version 0.10 (University of Amsterdam, Amsterdam, The Netherlands). Of the nine participants recruited, only eight completed the first day of the experiment as one of the participants had to leave unexpectedly for personal reasons. On the second day, one of the participants had a family emergency and was unable to participate in the study that day. Staff confirmed the reasons were unrelated to study protocol and treatment. An intention to treat analysis was performed. Data are reported as mean (SD) and mean change 95% confidence intervals (CI). Effect size is presented as partial eta squared throughout the paper. Effect sizes are interpreted as small (*n*^2^ = 0.01), medium (*n*^2^ = 0.09) and large (0.25). Alpha level for statistical significance was set a priori at ≤0.05.

## 3. Results

Our participants presented to the study being 21 ± 2 y of age, 1.77 ± 0.07 m tall, weighing 80.13 ± 13.18 kg and having a BMI of 25.60 ± 3.44 (kg/m^2^). The highest level of education obtained by all except two participants was ‘high school graduate’. One participant had ‘some high school, no diploma’ and another had ‘some college credit, no degree’. Average values for self-reported sleep duration were 8.1 ± 1.2 h/night, hours playing LoL was 10.3 ± 2.1 h/d, hours playing other video games was 1.8 ± 2.8 h/d, and exercise frequency was 4.2 ± 1.7 d/wk. An open-ended self-assessment of diet quality suggested that only four of the participants felt they consumed a healthy diet. All participants were found to regularly consume Reload prior to practices and competition.

**Figure 2:**
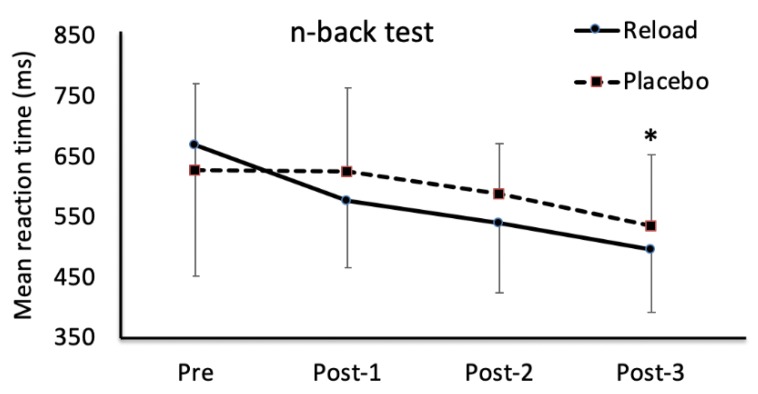
Mean (95% confidence intervals (CI)) reaction time for n-back test for Reload and placebo. * indicates a significant decrease for Reload at Post-3 compared with pre time point (*p* = 0.004).

An improvement was seen in the average reaction time for the n-back test for Reload from PRE (668.9 ± 216.3 ms) to POST3 (497.5 ± 105.1 ms, *p* = 0.004) (Figure 2), but there were no other significant differences across treatments or times (Table 1). When comparing between PRE and POST1 only, hand grip strength on the left hand (HGS-L) was higher for Reload at POST1 (35.0 ± 5.7 vs. 38.9 ± 8.3 kg, *p* = 0.037), but no other significant differences were seen between PRE and POST1. A separate analysis for day (visit 1 vs. visit 2) showed statistically significant improvements at visit two for n-back percentage correct (77.9 ± 12.7% vs. 86.8 ± 11.1%, *p* = 0.022) and mean reaction time (706.1 ± 154.3 ms vs. 603.8 ± 175.6 ms, *p* = 0.009) at PRE, indicating a possible learning effect.

## 4. Discussion

We hypothesized that the use of an energy drink would improve markers of cognitive and physical performance. We also hypothesized that mental fatigue accumulated through three consecutive video games would result in a decline in performance across a battery of cognitive and physical tests, and that the use of an energy drink would attenuate this decline. Our data suggest that playing three consecutive LoL games does not result in an accumulation of mental fatigue and the consumption of an energy drink did not improve measured performance parameters. Therefore, we rejected our research hypotheses.

There are several potential reasons for the lack of effects observed. One reason may be that the study participants play an average of more than 12 h of video games daily, suggesting more games may be required to accumulate a measurable amount of mental fatigue. Also, the study participants compete at the elite level and may be uniquely resistant to mental fatigue accumulation compared with recreational players. It is also possible that the chosen tests were not sensitive to the type of fatigue that may be accumulated during simulated eSport competition. However, we did not assess habitual caffeine intake, so it is possible participants may have been habituated to caffeine prior to initiating the study. Finally, our sample size was also low and since this study was novel, we could not conduct a power analysis so our study could have been under powered.

Another reason for the minimal observed effects may have been the relatively low dose of caffeine in the energy drink (150 mg; 1.9 ± 0.3 mg per kg body mass). Although we did not assess for habitual caffeine intake, our sample consisted of individuals who regularly consume energy drinks and may, therefore, have been habituated to the low caffeine dose. This dose was used because Reload is a single serving, commercially available drink that is commonly consumed by the athletes in and around competition. A wide range of caffeine doses have been used to study cognitive benefits, ranging from 50 mg [34] to 600 mg [35]. Several studies have looked at the combination of caffeine and theanine on mood and cognition and have found improved alertness [12,36], while others have reported improvements in cognitive performance but not subjective alertness [37].

Another ingredient in Reload that may offer cognitive benefits is alpha-GPC, a form of choline. A study looking at dietary choline intake and neurophysiological markers of attentional control in overweight adults found that higher choline intake was associated with more efficient neural processing as measured by brain activity—although, similarly to our study, there were no differences in performance on the Erikson Flanker task [38].

We wanted to determine the effects of energy drink consumption prior to three consecutive LoL games on markers of mental fatigue. No differences were found between the placebo and Reload. Although the study was not powerful enough to detect a difference between conditions, working memory was found to be improved within the Reload group. This is in accordance with previous work that has found improvements in working memory in healthy young volunteers who consumed an energy drink [39]. Although efforts were made to simulate the competition environment, the stakes were lower and the pressure to perform may not have been felt in the same way. It would be valuable for future research to consider pre- and post-testing during an in-season competition to determine if there are greater signs of mental fatigue.

In the present study, we also sought to characterize elite LoL athletes. Our sample had an average age of 20.8 years and average BMI of 25.6. This age is within the average age seen for peak fluid intelligence and information processing speed [40]. The average BMI falls within the ‘overweight’ category, but participant BMI ranged from 20.4 to 30.3. As previous work has linked LoL performance with fluid intelligence, future studies may examine the relationship between BMI and eSports performance [5]. Our data indicate that elite LoL athletes make sleep and practicing LoL a priority (sleeping eight hours a night and playing 10 h of LoL a day). This is significantly higher than the 5.28 h spent training daily found in a sampling of elite e-sports athletes from multiple games [41]. This may be due to LoL being more lucrative and competitive than other games. Furthermore, an exercise frequency of 4.2 times per week exceeds that of the average American. According to the Gallup–Sharecare 2017 State of American Wellbeing Report, only 55% of Americans are exercising at least three times per week [42]. The lifestyle deficit most apparent seems to pertain to nutrition. Only four of nine players provided a positive self-assessment of their diet, though the self-assessments themselves are short and brief. One participant indicated ‘I haven’t ever thought about it.’ Dietary education and intervention may prove to be a useful strategy for elite LoL players. However, research will need to be done to determine what dietary interventions, if any, are associated with performance improvements. A theoretical link can be made between an improved quality of diet and decreased adiposity and chronic low-grade inflammation on the one hand, and improved fluid intelligence and performance on the other [43].

A strength of our study is that, to our knowledge, this is the first study to examine the effects of mental and physical characteristics relative to (1) eSport performance concurrent with (2) the effects of ergogenically formulated nutrition supplementation on professional participants. Nevertheless, there are several limitations associated with our study. First, we tested professionals rather than amateurs. It is reasonable to suggest that professionals have greater motor skills and familiarity with the rigors of gaming. Second, we used a convenience sample of participants competing on the same team who agreed to participate using both treatment conditions. This, in turn, lends itself to a more controlled environment as the team trains under the same relative conditions. Third, due to the lack of similar research, we were unable to perform sample size estimates on our study populations; thus, the sample size is relatively small. Fourth, we examined a best-out-of-three gaming scenario and longer events (best of five) may induce a greater degree of overall fatigue whereby professional players are more fatigue resistant. Furthermore, it has been shown that mental fatigue accumulates throughout the day [44]. Future studies should examine if time of day influences performance, and if an energy drink beverage may induce enhanced performance improvements in the evening, when mental ability is decreased. It should be noted that the team orientation to a cumulative effort during competition lends itself to the potential for lower variance as opposed to examining individual players across multiple teams. Measures of mental fatigue did not accumulate over three games of LoL, nor did supplementation with an ergogenic aid targeting performance increases appear to have a benefit, except for working memory. Finally, we are unable to generalize our findings to amateurs or female contestants. More work needs to be performed using larger sample sizes and measures denoting “performance” parameters. It may also prove beneficial to investigate if BMI is related to performance, or if dietary education and intervention can enhance performance.

## 5. Conclusions

In conclusion, three games of LoL do not result in an accumulation of mental fatigue in elite LoL players. Further, the administration of a supplement designed to improve performance demonstrated no ergogenic effects relative to the indices examined in this study. It is unclear whether improvements in these measures may be associated with improved in-game performance. The present study provides suggestive information that can guide future studies in eSports. 

## Figures and Tables

**Table 1:** Test characteristics of the study participants. RT: Reaction Time. Data are mean (SD).

Test	Before Gaming		Post Game 1		Post Game 2		Post Game 3		Interaction	Statistical Effects		
	Placebo	Reload	Placebo	Reload	Placebo	Reload	Placebo	Reload		F	P_Bonf_	η² _p_
Flanker Congruent RT (ms)	363.25	367.63	381.88	350.88	404.75	332.50	352.88	348.75	Group	2.833	0.143	0.321
	62.55	48.72	81.17	38.16	62.39	21.84	42.13	32.13	Time	0.635	0.602	0.096
									G ×T ^1^	2.437	0.098	0.289
Flanker Opposite correct RT (ms)	429.50	462.50	394.88	377.38	414.63	382.75	391.50	397.13	Group	0.205	0.667	0.033
	106.35	196.68	64.62	22.46	46.71	49.29	61.76	46.23	Time	1.500	0.248	0.200
									G × T	1.317	0.300	0.180
Flanker Congruent Correct (%)	100.00	100.00	97.92	86.11	100.00	100.00	97.92	100.00	Group	0.010	0.927	0.002
	0.00	0.00	5.89	7.58	0.00	0.00	5.89	0.00	Time	3.602	0.046	0.474
									G × T	0.442	0.727	0.099
Flanker Opposite Correct (%)	88.73	93.96	88.64	93.35	96.81	91.14	94.77	90.93	Group	0.008	0.932	0.002
	9.37	6.98	10.47	7.85	4.46	8.15	7.49	11.53	Time	1.341	0.307	0.251
									G × T	1.498	0.265	0.272
Go-No-Go RT (ms)	350.68	318.36	352.96	321.42	322.58	312.02	335.80	317.19	Group	3.303	0.119	0.355
	38.63	52.99	52.99	44.53	52.81	33.10	41.00	31.30	Time	1.339	0.293	0.182
									G × T	0.331	0.803	0.052
Go-No-Go Correct (%)	92.33	90.11	92.07	91.67	88.22	92.23	92.99	88.27	Group	0.334	0.588	0.063
	5.65	13.11	8.13	16.06	12.16	13.11	6.31	12.03	Time	0.546	0.658	0.098
									G × T	0.679	0.578	0.120
n-Back RT (ms)	628.25	668.88	626.25	577.75	590.03	539.88	536.00	497.50	Group	0.068	0.803	0.001
	143.65	216.27	137.36	110.20	81.19	114.22	118.34	105.06	Time	4.304	0.019	0.108
									G × T	2.570	0.086	0.065
n-back Correct (%)	84.16	81.56	85.66	88.44	80.47	86.14	86.60	92.55	Group	0.056	0.82	0.009
	10.65	15.86	9.86	8.53	8.86	13.36	10.04	6.62	Time	2.872	0.065	0.324
									G × T	1.169	0.349	0.163
Hand Grip Strength Left Hand (kg)	33.96	36.44	34.21	38.89	33.45	36.73	33.70	36.63	Group	4.357	0.082	0.421
	6.88	8.00	5.86	8.29	5.27	6.30	5.44	7.11	Time	1.875	0.170	0.238
									G × T	2.299	0.112	0.277
Hand Grip Strength Right Hand (kg)	37.21	38.91	37.78	39.51	38.48	39.28	38.28	41.25	Group	0.887	0.378	0.112
	6.95	7.01	6.20	6.86	7.72	6.53	6.99	7.13	Time	0.417	0.539	0.056
									G × T	0.122	0.738	0.017
Tap Left (n)	70.89	73.50	74.67	71.75	74.00	71.50	71.63	72.88	Group	0.204	0.667	0.033
	9.39	6.39	15.54	9.04	7.73	7.75	5.42	6.56	Time	0.988	0.421	0.141
									G × T	1.665	0.210	0.217
Tap Right (n)	74.89	75.50	82.00	75.75	78.88	76.00	78.38	77.25	Group	2.118	0.196	0.261
	9.01	8.09	10.34	8.55	7.74	8.64	7.50	9.21	Time	1.792	0.185	0.230
									G × T	1.581	0.229	0.209

^1^ G × T: group × time interaction.
